# A laboratory protocol for shoulder-head and head-ground dummy head accelerations during player high-speed rugby tackles

**DOI:** 10.7717/peerj.20953

**Published:** 2026-03-25

**Authors:** Elizabeth J. Bradshaw, Alex Conte-Biggar, Eric J. Drinkwater, Bradley A. Morris, Lyndell M. Bruce, Patria A. Hume, Doug A. King

**Affiliations:** 1 Centre for Sport Research, Institute for Physical Activity and Nutrition, Deakin University, Melbourne, Victoria, Australia; 2 Sports Performance Research Institute New Zealand (SPRINZ), Auckland University of Technology, Auckland, New Zealand; 3 Department of Physiotherapy, Division of Allied Health, Austin Hospital, Melbourne, Victoria, Australia; 4 School of Science and Technology, University of New England, Armidale, New South Wales, Australia; 5 Auckland Bioengineering Institute, University of Auckland, Auckland, New Zealand; 6 Tech & Policy Lab, Law School, University of Western Australia, Perth, Western Australia, Australia; 7 Traumatic Brain Injury Network, Auckland University of Technology, Auckland, New Zealand; 8 Wolfson Research Institute for Health and Wellbeing, Durham University, Durham, England, United Kingdom

**Keywords:** Biomechanics, Concussion, Football, Head acceleration, Injury, Protective equipment, Collision, Method

## Abstract

**Background:**

The purpose of this study was to demonstrate the effectiveness of a novel laboratory testing protocol for dummy head biomechanics of the shoulder-head and subsequent head-ground impacts during rugby tackles. Currently, no research in tackles utilizes a real player as the tackler and considers the second impact when the opponent’s head hits the ground.

**Methods:**

A dummy was instrumented with an inertial measurement unit (IMU; 1,200 Hz) behind the right ear. Eleven rugby players with a shoulder placed IMU executed right shoulder high tackles to the left side of the dummy’s head at two closing velocities (high-speed 15–17 km/hr and very-high-speed 21–23 km/hr) for three dummy head conditions (no headgear, club-level headgear, professional-level headgear). Peak resultant linear and rotational accelerations were calculated for the first impact event (shoulder-head) for the player’s shoulder IMU and for the two impact events (shoulder-head, head-ground) for the dummy head IMU.

**Results:**

Whilst the player experienced low linear accelerations (fast-speed = 10 g, very-fast-speed = 13 g) through their shoulder during the tackle impact (shoulder-head collision), the linear accelerations were six times higher (63 g; *p* = 0.003) for the dummy head for the high-speed approach and seven times higher (88 g; *p* = 0.003) for the very-high-speed approach. The second head-ground impact was generally lower for the linear accelerations (*p* < 0.004) but unchanged for the rotational accelerations (fast-speed = 4,589–4,955 rad/s^2^, very-fast-speed = 6,948–7,123 rad/s^2^) for the dummy head. Resultant rotational acceleration significantly increased for the dummy head-ground impact when club-level headgear was worn for the very-high-speed approach (8,434 rad/s^2^, *p* = 0.045). No other significant differences were observed between the no headgear and headgear tests.

**Conclusions:**

This study demonstrates the effectiveness of a protocol measuring linear and rotational accelerations of a dummy’s head during high-speed rugby tackles where a real player’s shoulder hit the dummy’s head and the dummy’s head hit the floor. Trialing the protocol showed high impact accelerations experienced when receiving a tackle did not reduce with headgear. The experimental methodology and tools developed provide the basis for more complete testing of head biomechanics in tackles.

## Introduction

Contact sports such as rugby pose an injury risk due to the highly physical nature of play, and high forces that are imparted onto athletes (King et al., 2018; Bradshaw et al., 2024; https://anzors.org.au/wp-content/uploads/2025/03/ANZORS-2024-ABC14-proceedings.pdf). Concussion is a subset of mild traumatic brain injury (mTBI) and is a commonly reported injury in rugby (league, union)^i^ (Gardner et al., 2014; King et al., 2019; West et al., 2021). Concussion is a complex pathophysiological process affecting the brain which is incited by traumatic biomechanical forces (indirect and/or direct) that causes brain “shaking” (Tamimi et al., 2025). Concussion accounts for 9% of all injuries within legal play in rugby but increases to 29 to 35% of all injuries when illegal gameplay or tactics are analyzed (Gardner et al., 2014; King et al., 2018; Bradshaw et al., 2024). High shoulder tackles to an opposition player’s head are an example of an illegal tackle that is still observed in match-play, whether purposeful or accidental, and contributes to these injury statistics (McIntosh, 2005; Gardner et al., 2014; Tucker et al., 2017; Raftery, Tucker & Falvey, 2021; Bradshaw et al., 2024). In fact, the highest incidence of rugby concussion injury was a result of shoulder-to-head collisions (35%) during tackles and game play (Gardner et al., 2014; Rock, 2016). Concussion injury from these shoulder-to-head tackles (Rock, 2016) has been reported to predominantly affect the opponent (ball carrier/tacklee (King et al., 2010; King et al., 2014; Gardner et al., 2015)); but also can affect the tackler (Gardner et al., 2014; Cross et al., 2019). Further, it has been recently demonstrated that concussion risk increases with sanction severity for illegal high tackles for both tacklers and ball carriers. It was reported that there is a 272-fold increased concussion risk for red-carded tackles when compared with legal tackles, and a six-fold increased concussion risk for red-carded tackles than yellow-carded tackles (Tucker et al., 2024). The player with the lower momentum, or standing still, also has a substantially higher risk of injury in high-speed tackles due to the differential speed conditions (Garraway et al., 1999). Aside from specific match rules and infringement penalties (including match review panels), various strategies have been employed to lower the risk of head injury during rugby match-play. These include the use of headgear (extrinsic strategy) and increasing player protective factors through pre-season preventative training (intrinsic strategy), such as training interventions to increase player’s neck strength, and safe tackling technical guidelines such as tackling low to avoid the trunk and head/neck, from an oblique (offset) angle instead of purely front-on, increased focus on training the non-preferred tackle side (Posthumus & Viljoen, 2008; Quarrie, 2008; Seminati et al., 2025).

Headgear must meet minimum World Rugby standards which includes soft materials with padding no thicker than one cm and density under 45 kg/m^3^ (World Rugby, 2026). Manufacturers also consider other factors such as cost for junior players and heat dissipation for professional players. This likely causes small variations in the design and thus mechanical performance of specific headgear models. Headgear may seem a logical precaution for reducing concussion, however, while the current use of soft, polyurethane headgear worn in rugby help reduce superficial injuries to the head and ears (haematoma, lacerations, and abrasions) they are not consistently supported by scientific evidence to reduce more serious head injuries such as concussion (AlAttar et al., 2024; Baron et al., 2020; Stokes et al., 2021; Henley et al., 2023). External headgear does not stop the brain from “shaking” and hitting the internal skull surface, nor can it stop the shearing of the brain structures (Henley et al., 2023).

Headgear is commonly assessed in mechanically controlled laboratory conditions *via* a single linear force (ballistic) impact, drop impact, or pendulum impact test on a Hybrid III head-neck system. This may not adequately represent the impact forces applied to the opponent’s head during gameplay as it uses a rigid impactor that does not fully replicate the combination of resultant linear and rotational velocity/acceleration of the head during a tackle collision (Wu, 2020). This combination of forces has the potential to affect concussion incidence and severity. This type of testing also doesn’t capture the second head impact if the player is knocked over and their head strikes the ground. This event is frequently present in on-field incidents and can lead to concussion (McIntosh et al., 2019). Player behaviour known as “risk compensation” may also influence the effectiveness of headgear. Risk compensation is a reactive form of play that involves more aggression to deliberately tackle harder when the opponent is wearing headgear (Hagel & Meeuwisse, 2004; McIntosh, Caponecchia & Usman, 2011; Brownbridge, 2020). This behaviour is difficult to identify, which therefore impacts on the findings of observation studies of head injuries during match-play. Therefore, using athlete-led tacklers and a full-body dummy may afford the opportunity to measure more realistic head acceleration data of the ball carrying opponent (tacklee).

Considering the limitations in engineering laboratory studies of tackling impacts, a test protocol with higher ecological validity would allow for greater exploration of factors that influence the safety of tackles resulting in impact of the opponent’s head. Therefore, the objective of this study was to develop and assess the reliability of a protocol for laboratory measurement of dummy head biomechanics from initial to ground impact during a dangerous rugby high tackle of the shoulder hitting the dummy head by a player. This study also aimed to show context validity by examining (i) the effect of tackler height and body mass on the head mechanics of the opponent (tackle) and (ii) the effect of tackler approach speed on the head mechanics of the opponent (tacklee) with and without headgear.

## Materials & Methods

### Design and ethics

The experimental laboratory-based study was conducted in the Biomechanics laboratory at Deakin University in Melbourne. Participants were asked to complete one testing session of up to two hours. Ethical approval from the Deakin University Human Research Ethics Committee (2019-158) was obtained prior to the study commencing.

### Participants

Eleven physically active male club-level (first division of local state competition) rugby union players from metropolitan Melbourne participated in the study (aged 18 to 41 years; height 1.83 ± 0.05 m; body mass 103.9 ± 10.1 kg). Participants were recruited *via* an email to local rugby union clubs, posters and word-of-mouth.

All volunteers were screened using two custom surveys to identify whether the participant met study inclusion criteria. Survey One included (i) a Pre-Participation Screening Survey that included the Exercise and Sports Science Australia (ESSA) Adult Pre-Exercise Screening Tool 2011 (Stage 1 only: Norton & Norton, 2011) (ii) screening questions to detect injury, illness or other health problems during the preceding week that interfered with normal training and/or competition (Clarsen et al., 2014), (iii) a Past and Current Sport Participation Survey (Roos, Brandsson & Karlsson, 2001), and (iv) past injury in the last 12 months (Finch, Valuri & Ozanne-Smith, 1999). Survey Two asked volunteers about their past and current sport participation.

All participants were able to confidently execute legal tackle technique with their right shoulder, were injury free and physically active; defined using the Australian standards of five hours of moderate intensity physical activity or two and a half hours of vigorous intensity physical activity per week (Brown et al., 2013); at the time of testing, had no history of shoulder, knee or ankle injury that required surgery or shoulder, knee or ankle injury in the previous 12 months, and provided informed written consent prior to participation in the study.

### Tackle dummy instrumentation

**Figure 1:**
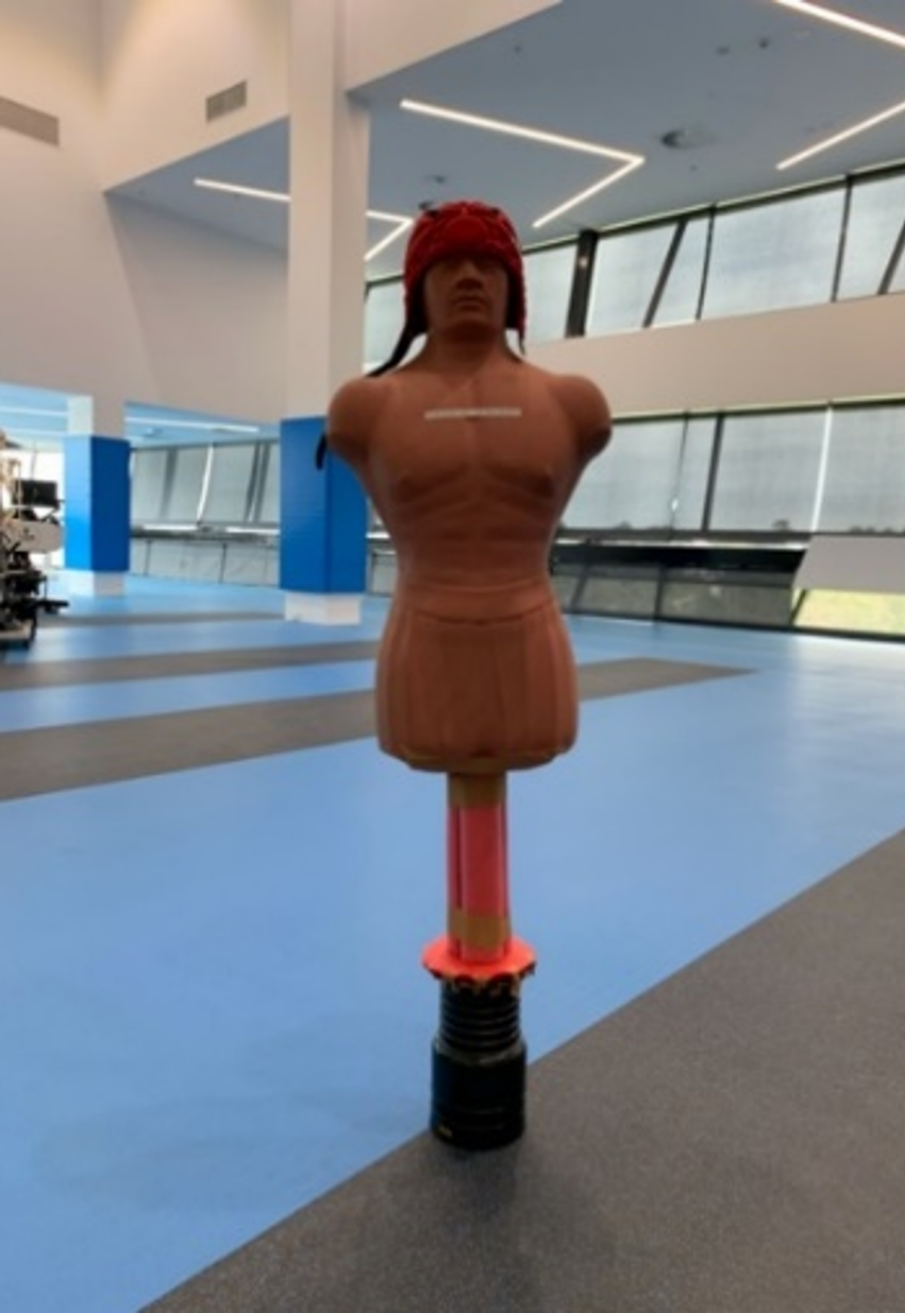
The modified body opponent bag (BOB) tackle dummy.

To enable laboratory measurement of linear and rotational accelerations of a dummy’s head during high-speed rugby tackles with shoulder-head and head-ground dummy impacts a free-standing tackle dummy (Body opponent bag (BOB), XL, 1.4 m, Shinobi, Australia) designed for martial arts and boxing was modified (Fig. 1). The tackle dummy consisted of a large, male-sized upper body without arms, fixed to the large, weighted base about a flexible column. The body contained a high strength ethylene vinyl acetate (EVA) bonded foam with a heavy spring core, designed to feel like a human body when struck. Importantly for this study, the neck was semi-rigid, and the neck moved when the head was struck. The stiffness of the dummy neck may replicate the neck strength of forwards (front-row players) at the senior elite level, but not younger players (Chavarro-Nieto et al., 2021). According to Walsh et al. (2018) the neck stiffness of the model may present a biased response for the acceleration components, but not for the resultant acceleration magnitudes (linear, angular) for shorter duration impacts. That bias due to the neck stiffness of the tackle dummy is consistent throughout the experimental tests. The original base was replaced using a custom-made aluminium base with foam padding that lowered the erect standing tackle dummy height from 1.67 m to 1.40 m to enable replication of an opposing player on the field who may be leaning forward in possession of the ball. The mass of the modified dummy was 16.60 kg. The tackle dummy was instrumented with a second-generation, inertial measurement unit (IMU; Blue Trident, Accelerometer Range = ±200 g, Gyroscope Range = ±2000^0^/s; Vicon, Oxford, United Kingdom, 1,200 Hz). For information on the frequency response of the IMUs, please see (Bosio et al., 2025). The sensor was secured onto the right side of the tackle dummy’s head, behind the ear (equivalent to the right mastoid; bony prominence behind the human ear), with rigid athletic strapping tape (Professional super rigid sports tape, 50 mm × 13.7 m; Victor Sports, Asquith, NSW, Australia). The athletic strapping tape was applied to the full circumference of the tackle dummy’s head to obtain rigid fixation of the IMU. Raw measures of angular velocity from the IMU gyroscope have previously been validated with an optical system by Walker et al. (2017).

### Procedure and data collection

Participant’s height and body mass were measured using a stadiometer (Stadi-O-Meter; Novel Products Inc, Rockton, IL, USA) and scales (HW-PW200; A&D Company Ltd, Tokyo, Japan).

One inertial measurement unit (IMU) was placed on the supraspinous fossa (cranial to the spine; Höglund, Grip & Öhberg, 2021) of the participant’s right scapula using rigid athletic tape to measure shoulder impact linear acceleration. This landmark is an easily identifiable bony landmark that enables consistent IMU placement in a position that does not impede the participants movement or create a safety risk by being in the zone of impact during the tackle. All IMU data (participant’s right shoulder impact and tackle dummy’s head impacts) were captured using an iPad (6th Generation; Apple, Cupertino, CA, United States) with the manufacturer’s application (app) software (CaptureU, Vicon, Oxford, United Kingdom).

Participants completed the Waterloo Footedness and Handedness questionnaires (Elias & Bulman-Fleming, 1998). All participants were right-handed and footed, except for participant eight who was left-footed. However, all participants stated their preferred side for tackling was the right side.

Participants completed a set warm-up of dynamic stretching and running that gradually increased in speed, with slow acceleration and deceleration under the direction of a strength and conditioning coach with experience working with club-level and professional rugby players.

The modified tackle dummy was positioned on the right side of participant at the end of a 10 m runway, with the left side of the dummy’s head facing the participant. Participants were asked to perform a legal tackle technique but executed high at or above the level of the shoulders, and to strike the left side of the dummy’s head with their right shoulder during a high tackle.

Participants did some practice runs through timing gates (Swift Speedlight Pro4; Swift Performance, Wacol, Queensland, Australia), with immediate feedback, to become familiar with the running speeds required. Participants used an approach distance of 10 m, ran in a straight line (linear approach), and achieved a final closing velocity no more than 10% above the 15 km/hr (4.17 m/s) and 21 km/hr (5.83 m/s) target speeds through the timing gates positioned in the final 1 m of the approach (9–10 m interval) for a successful trial to be recorded. These high-speed (>15 km/hr) and very-high-speed (>21 km/hr) running speeds were identified based on rugby league competition match analysis (Kempton, Sirotic & Coutts, 2014), which are similar to average closing speeds of 5.5. m/s (19.8 km/hr) reported for tacklers executing side-on tackles during varsity rugby union matches (Hendricks et al., 2012).

**Figure 2:**
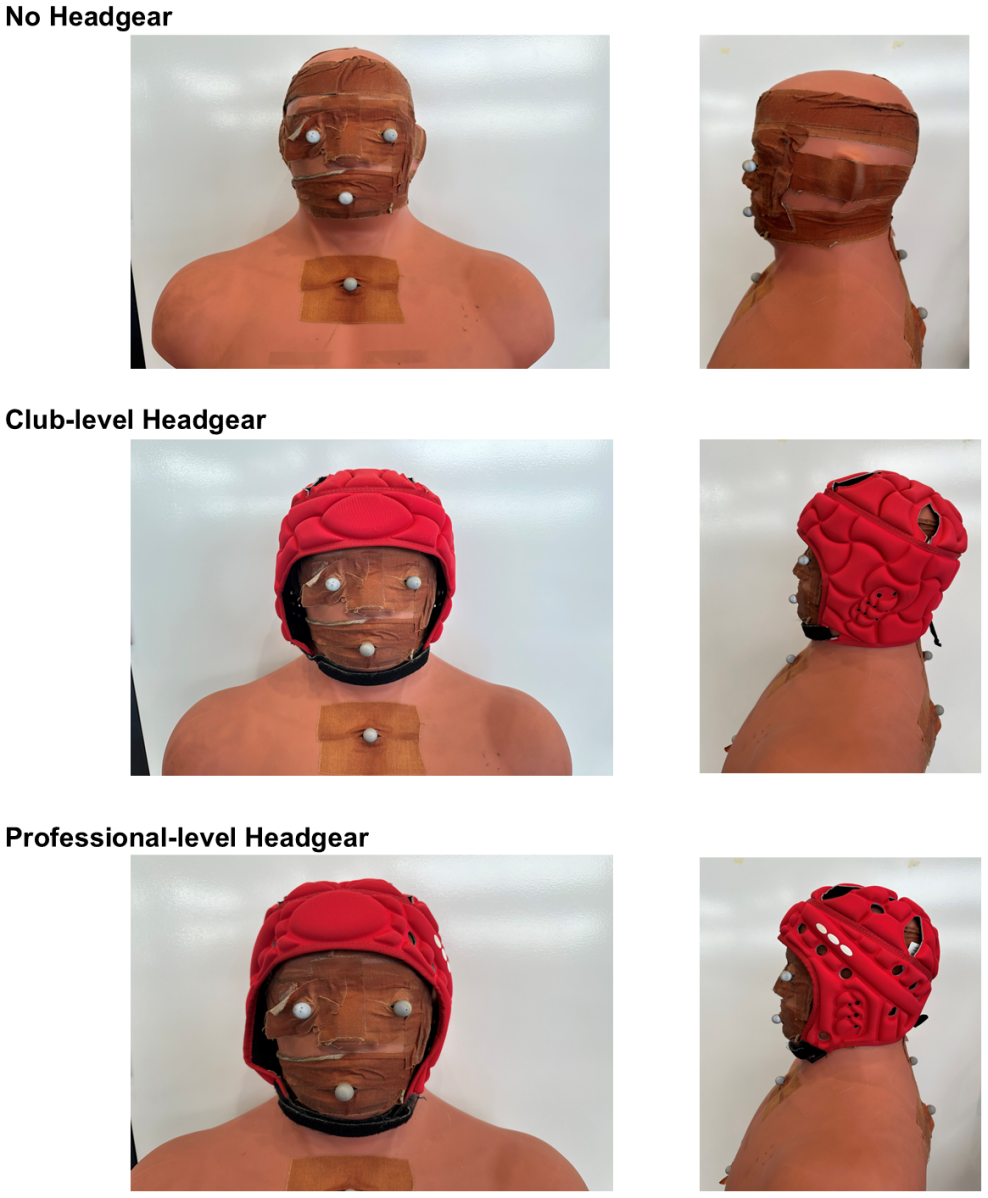
The headgear conditions tested during the tackles including no headgear, club-level headgear, and professional-level headgear. The headgear was purchased without the manufacturer’s logo. Each headgear was International Rugby Board (IRB) approved.

Three conditions were used to test the protocol (Fig. 2): (1) No headgear, (2) International Rugby Board (IRB) approved club-level headgear by Club Plus Headgear and (3) IRB approved professional-level headgear by AIRFLOW (both purchased from Canterbury, New Zealand). For the two headgear conditions the headgear was tightly fastened using the strap under the chin.

Participants completed three trials of the two approach speeds and three conditions (*i.e.,* 18 trials) given skills that involve power and speed require three trials for accurate measurements (Moir et al., 2004). A trial was considered successful when participants ran within +0–10% of the prescribed running approach speed and tackled the dummy with contact of their right shoulder to the dummy’s head during the tackle. The tackler did not hold on to the dummy and only the dummy could fall to the ground. This protocol therefore examined both initial contact dynamics and subsequent ground impact following separation. While tacklers commonly maintain contact through to ground, separation post-initial contact occurs frequently in modern rugby, particularly in scenarios involving head contact. This type of tackle is dangerous in contemporary rugby, making our experimental conditions highly relevant. Participants were asked to rest for a self-selected period after each running approach, and for 5 min between conditions. Trial order was randomized between participants, so that each participant in the study completed a different combination of approach speed and headgear conditions.

### Data analyses

IMU captured data included linear accelerations (*a*_*x*_, *a*_*y*_, *a*_*z*_) and rotational (angular) velocities (*ω*_*x*_, *ω*_*y*_, *ω*_*z*_). Linear accelerations were converted from m/s^2^ to gravitational units (g) by dividing these measures with respect to gravity (−9.81 m/s^2^). Rotational velocity measures were converted into rotational acceleration (*α*_*x*_, *α*_*y*_, *α*_*z*_). using the first central difference method, using two consecutive velocity vectors, and converted from degrees/s^2^ to radians/s^2^ (multiplication by *π*/180) for consistency with previous published reports (King et al., 2018). Resultant linear (*a*_*r*_) and rotational (*α*_*r*_) acceleration magnitudes were obtained as the magnitude of the three orthogonal components:

\begin{eqnarray*}{a}_{r}=\sqrt{{a}_{x}^{2}+{a}_{y}^{2}+{a}_{z}^{2}}~~~~~~~~~~~~{\propto }_{r}=\sqrt{{\mathop{\propto \nolimits }\nolimits }_{x}^{2}+{\mathop{\propto \nolimits }\nolimits }_{y}^{2}+{\mathop{\propto \nolimits }\nolimits }_{z}^{2}}. \end{eqnarray*}

**Figure 3:**
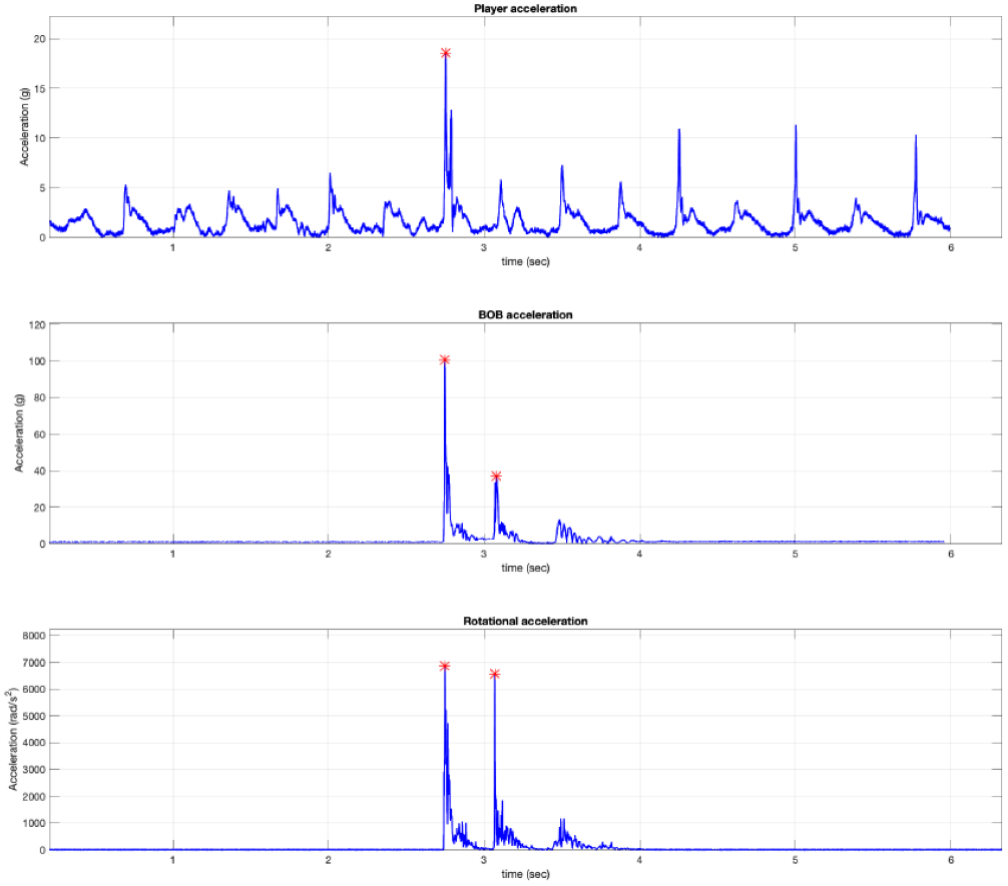
Example graph outputs from the MatLab program with the impact peaks identified for the player’s shoulder IMU and the tackle dummy’s head IMU.

Player and tackle dummy accelerations were analyzed using a custom data processing program (MatLab version R2019a; MathWorks Inc., Natick, MA, USA). Matlab was used to graph data and automatically identify the expected two peaks coinciding with the shoulder-head impact and head-ground impact (Fig. 3). Trial data for each approach speed and condition were averaged across the three trials for each player.

The group data set was imported into statistical software (version 25; SPSS, IBM, Armonk, NY, USA). All statistical analyses were set at an alpha level of 0.05. Data normality was tested using the Shapiro Wilk test, revealing many measures in the dataset were not normally distributed, hence non-parametric tests were used. Descriptive statistics were obtained (median (MED), inter-quartile range (IQR)).

Relative and absolute statistical tests were used to assess inter-trial reliability for the baseline tests (no headgear condition; Atkinson & Nevill, 1998). Friedman’s analysis of variance (ANOVA), intra-class correlation coefficient (two-way mixed (2), absolute agreement (k)), Hedge’s g effect size (ES), and the average difference in the mean (MDiff%) were conducted. The criteria for reliability for each measure were Friedman’s *p* ≤ 0.05, ICC ≥ 0.75 (Koo & Li, 2016), ES < 0.60 (trivial to small effect) (Hopkins, 2006), and MDiff% ≤ 10.00. The interpretation for overall reliability was ‘very good’ when all criteria were met, ‘good’ when three criteria were met, ‘moderate’ when two criteria were met, and ‘poor’ when less than two criteria were met (Campbell et al., 2020).

Spearman’s Rank-Order Correlation analysis was utilised to identify any relationships between player anthropometry (height, body mass) on the impact biomechanics measures for the no headgear condition. Scatter plots were created for any significant correlations to confirm a monotonic relationship. If a monotonic relationship was not evident, a Kendal’s tau b was run to confirm the relationship.

Friedman analysis was used to identify if there was a main effect of headgear condition (Moir et al., 2004; Marshall et al., 2005), followed by Wilcoxon tests to test pairwise differences. Wilcoxon tests were also used to identify differences in the impact biomechanics measures due to tackler approach velocity, and also between the two impact events.

Finally, to aid in contextual interpretation of the measures in this study, the head impact biomechanics results for the two approach speeds and two head-impact events were then classified for likelihood of concussion (LOC) injury. A classification system for adult male football players (Australian rules football and rugby league codes) of 50% LOC if greater than 65.1 g/1,747 rad/s^2^, and 75% LOC if greater than 88.5 g/2,296 rad/s^2^ was used (McIntosh et al., 2014).

## Results

**Figure 4:**
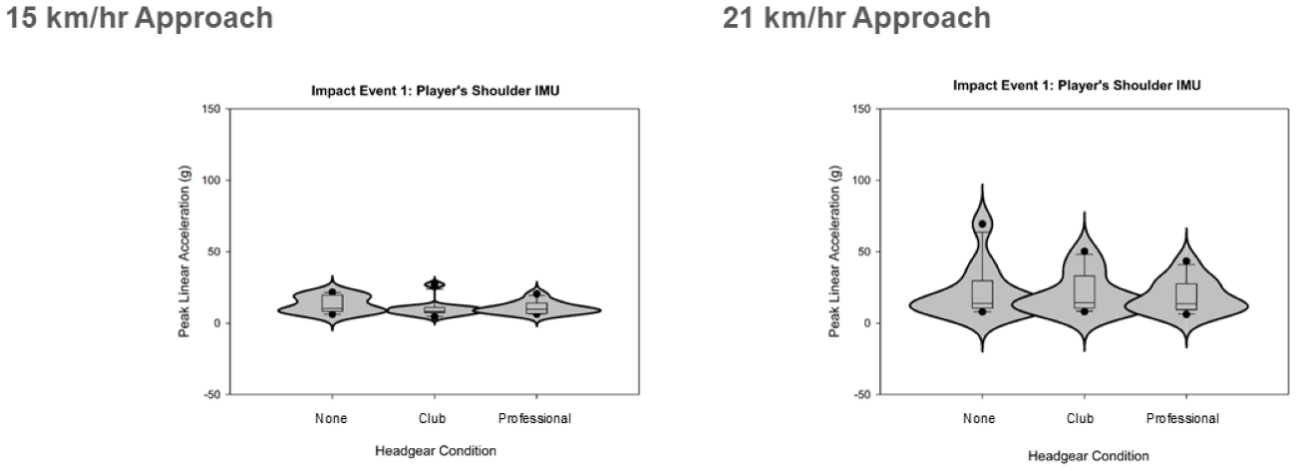
Violin plots displaying the resultant peak linear accelerations recorded at the participants shoulder during the tackle for the two approach speeds and three conditions (two with headgear).

**Figure 5:**
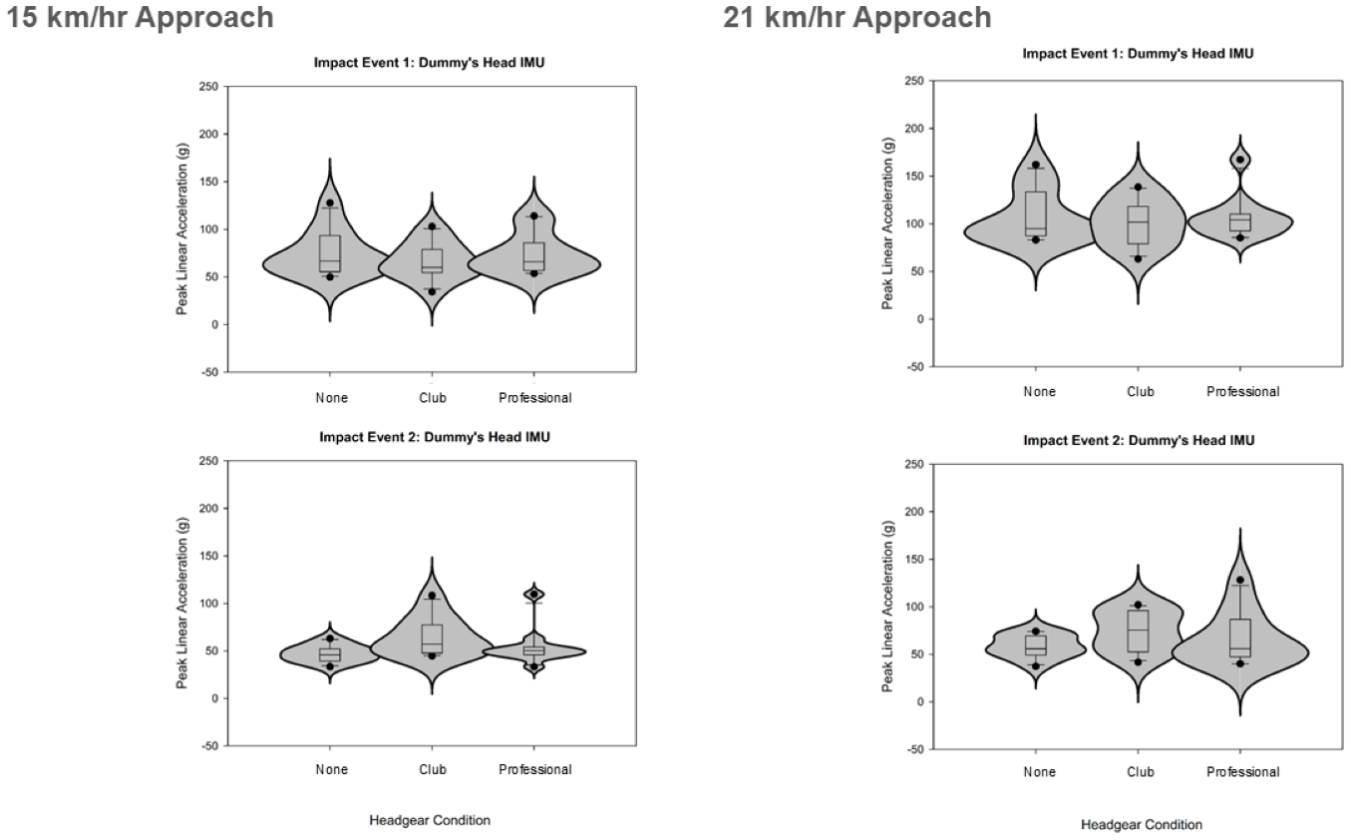
Violin plots displaying the resultant peak linear accelerations recorded at the tackle dummy’s head during the (1) shoulder-head impact and the (2) head-ground impact events. The tackles were tested for the two approach speeds and three conditions (two with headgear).

**Figure 6:**
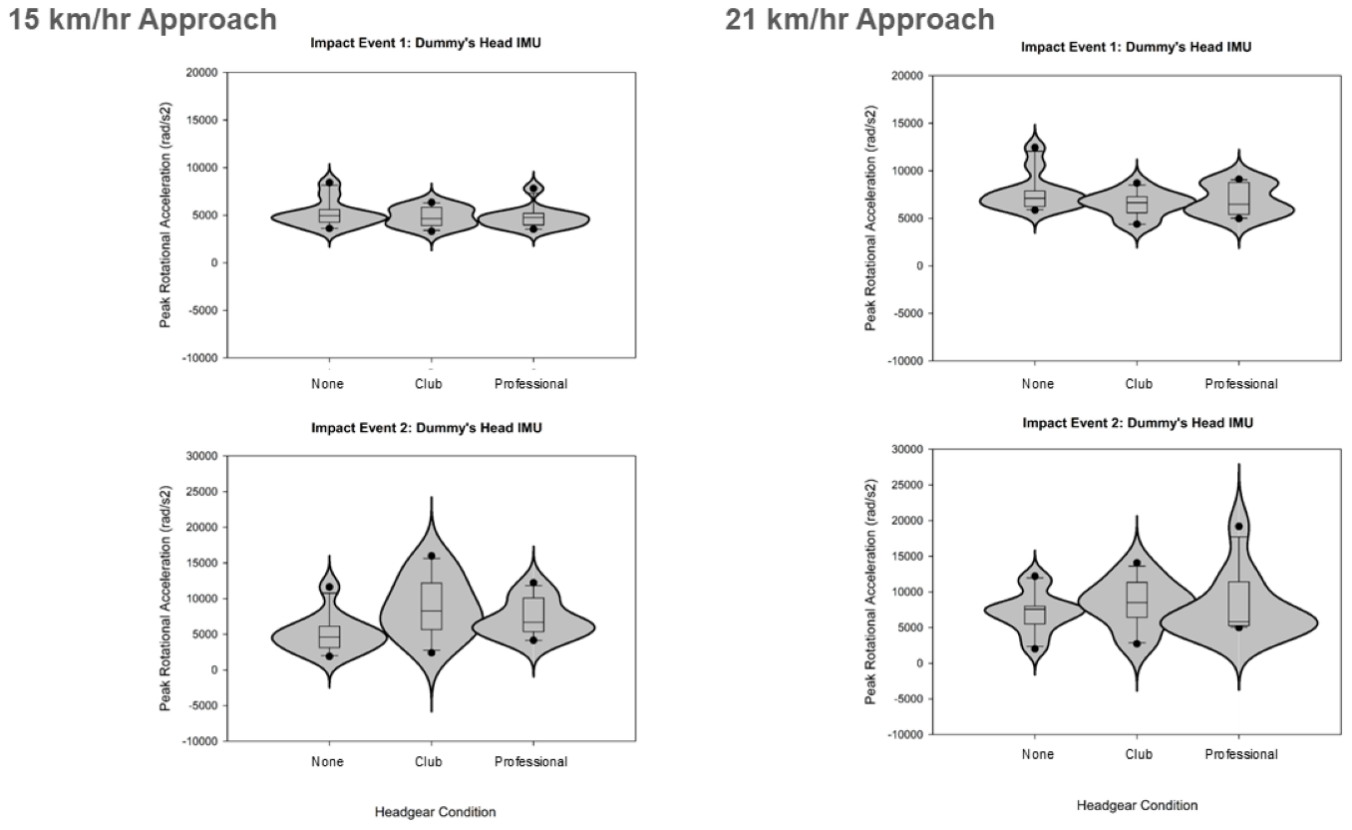
Violin plots displaying the resultant peak rotational accelerations recorded at the tackle dummy’s head during the (1) shoulder-head impact and the (2) head-ground impact events. The tackles were tested for the two approach speeds and three conditions (two with headgear).

The protocol testing with two closing approach speeds and three headgear conditions collected data for peak linear accelerations of the player’s shoulder, and peak linear and rotational accelerations of the dummy’s head during high-speed rugby tackles with shoulder-head and head-ground dummy impacts (Figs. 4–6).

### Inter-trial reliability

The inter-trial reliability results are summarized in Table 1. Good to Very Good reliability was identified for all tackling biomechanic measures for the *high-speed* trials. Poor to Very Good reliability was identified for the tackling biomechanic measures for the *very-high-speed* trials.

### Baseline tackle biomechanics

#### Tackler

Tackler height and body mass generally had no significant effect on the tackle impact biomechanics, with two exceptions. Tackler height influenced the dummy head linear acceleration during the second, head-ground impact (*r*_s_(11) = −0.732, *p* = 0.010; *τ*_b_ = −0.623, *p* = 0.009) for the *very-high-speed* trials. Tackler body mass influenced the tackler’s shoulder linear acceleration (*r*_s_(11) = −0.700, *p* = 0.016; *τ*_b_ = −0.564, *p* = 0.026) for the *high-speed* trials but not for the *very-high-speed* trials.

The Wilcoxon tests identified significantly higher tackler’s shoulder linear acceleration (*Z* = 2.134, *p* = 0.033) for the *very-high-speed* trials. The median shoulder linear acceleration was low (*high-speed* 10 g; *very-high-speed* 13 g) but varied amongst players from 6 to 22 g for the *high-speed* and 8 to 69 g for the *very-high*-speed approaches.

#### Dummy opponent’s head

The dummy head linear acceleration was significantly higher during the first, shoulder-head impact (fast-speed: 62.84 g → 51.45 g, *Z* =  − 2.845, *p* = 0.004; very-fast-speed: 87.77 g → 49.16 g, *Z* =  − 2.936, *p* = 0.003) whereas the dummy head rotational acceleration was not significantly different between the two impact events (fast-speed: 4,955.13 rad/s^2^ → 4,589.58 rad/s^2^; very- fast-speed: 6,948.36 rad/s^2^ → 7,122.72 rad/s^2^).

**Table 1:** Intrasession reliability of the IMU measures of the tackle impact events for the two approach (closing) speeds.

Measure	Criteria	15 km/hr approach: high-speed					21 km/hr approach: very-high-speed				
		Shoulder-head collision			Head-ground collision		Shoulder-head collision			Head-ground collision	
		Player’s shoulder IMU PLA (g)	Tackle dummy’s head PLA (g)	Tackle dummy’s head PRA (rad/s^2^)	Tackle dummy’s head PLA (g)	Tackle dummy’s head PRA (rad/s^2^)	Player’s shoulder IMU PLA (g)	Tackle dummy’s head PLA (g)	Tackle dummy’s head PRA (rad/s^2^)	Tackle dummy’s head PLA (g)	Tackle dummy’s head PRA (rad/s^2^)
Friedman p	≤0.05	0.236	0.741	0.497	0.497	0.497	0.641	0.741	0.497	0.020	0.497
ICC (2, k)	≥0.750	0.759	0.875	0.873	0.576	0.877	0.705	0.863	0.863	0.584	0.683
Hedge’s g	<0.600	0.358	0.218	0.431	0.190	0.209	0.370	0.013	0.022	0.591	0.148
MDiff%	≤10.00	18.75	7.14	12.69	4.78	11.79	22.81	0.39	0.57	16.62	7.92
Reliability rating		Good	Very good	Good	Good	Good	Poor	Very good	Very good	Moderate	Good

**Notes.**

p, probability of a statistically significant difference between trials; ICC, intra-class correlation; MDiff%, the mean difference percentage between trials; IMU, inertial measurement unit; PLA, resultant peak linear acceleration; PRA, resultant peak rotational (angular) acceleration; km/hr, kilometres per hour; g, gravitational units where 1 g is 1 × −9.81 m/s^2^; rad/s^2^, radians per second squared; Very Good, four criteria was met; Good, three criteria were met; Moderate, two criteria were met; Poor, one or zero criteria were met.

### The effect of headgear on the tackle biomechanics

Significant differences were identified between headgear conditions for the second, head-ground impact and for the *very-high-speed* approach only. For resultant linear acceleration (*χ*^2^(2,11) = 6.727, *p* = 0.035) the club-level headgear had the highest mean rank (2.64), followed by no headgear (control; 1.73), and professional-level headgear had the lowest rank (1.64). For resultant rotational acceleration (*χ*^2^(2,11) = 8.909, *p* = 0.012) the club-level headgear had the highest mean rank (2.45), followed by professional-level headgear (2.27), and no headgear (control) had the lowest rank (1.27).

### Likelihood of concussion classification

For the *high-speed* tackles the peak linear accelerations during both impact events were rated at less than 50% likelihood of concussion (LOC) injury (McIntosh et al., 2014), regardless of whether headgear was worn or not, and for both impact (collision) events. Whereas the peak rotational accelerations during both impact events were generally associated with a greater than 75% likelihood of concussion (LOC) injury, regardless of whether headgear was worn or not.

For the *very-high-speed* tackles the peak linear acceleration for the no headgear condition was rated as 50 to 74% LOC, and greater than 75% LOC injury for the club- and professional-level headgear for the shoulder-head impact. During the head-ground impact the LOC risk was less than 50% for all headgear conditions. However, the peak rotational accelerations during both impact events were associated with a greater than 75% LOC injury for all headgear conditions tested (no headgear, club- and professional-level headgear) (McIntosh et al., 2014).

**Figure 7:**
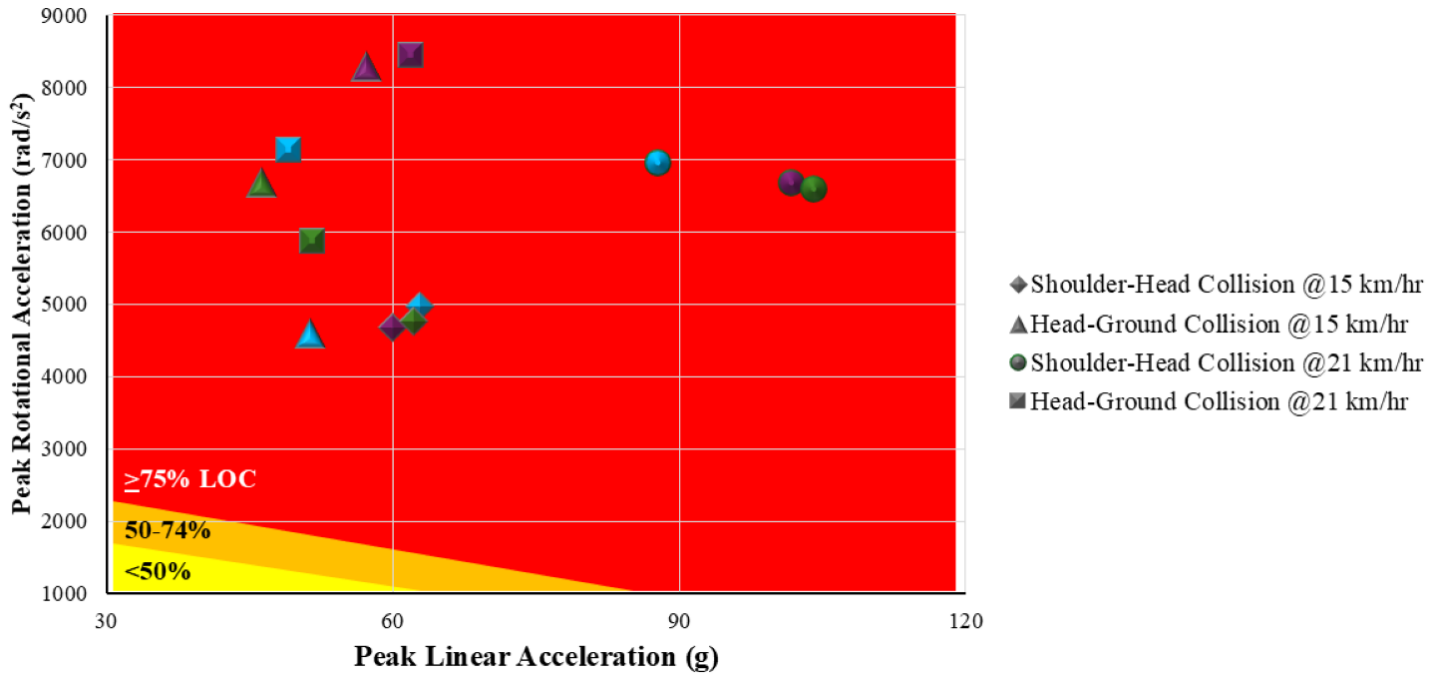
Likelihood of conclusion (LOC) risk matrix based on the measured peak linear and peak rotational accelerations for the tackles tested. Classifications were based on previously reported thresholds (McIntosh et al., 2014). Points colour coded as follows: Blue = No headgear (Control); Purple = Club-level headgear; Green = Professional-level headgear.

When accounting for all factors (closing speed, headgear condition, linear & rotational acceleration) the LOC injury was generally greater than 75% during the controlled tackles onto the dummy in the laboratory tests (McIntosh et al., 2014) (Fig. 7).

## Discussion

Brain injuries occur from a combination of linear and rotational acceleration. Some early research, highlighted by Post & Hoshizaki (2012), used a device that applied rotational accelerations to the heads of monkeys and resulted in mild (concussion) to severe acquired brain injury (ABI) and even death (Adams, Graham & Gennarelli, 1981; Gennarelli et al., 1982; Post & Hoshizaki, 2012). For ethical reasons, on-field impacts have since been recreated in laboratories. The simplest human surrogate for head impact testing is a head form or dummy head which can be unconstrained at impact when dropped (*e.g.*, equestrian helmet tests), launched (*e.g.*, vehicle accident testing), or hung and struck (Funk et al., 2022). The sophistication of the human surrogate varies and even the Hybrid III has limitations when used to simulate real-world head impacts, such as their fixed shape and size and that they don’t match a tacklee’s pre-impact posture (Funk et al., 2022). Further, most research only recreates a single impact, instead of attempting to reconstruct the two (or multiple) impact events often seen in incidences concussion during match-play (Funk et al., 2022). When recreating the impact, as many biomechanical parameters such as the approach closing velocity and the impact location, should be tested as closely as possible (Funk et al., 2022). In this study, a more ecologically valid method of testing head impact biomechanics when receiving a tackle was developed than has been previously used. Our approach enabled, for the first time, the biomechanics of this tackle to be assessed from a player tackling a dummy to measure the shoulder-head impact and the head-ground impact of the dummy. This novel testing revealed that the linear acceleration was higher during the shoulder-head impact than the head-ground impact (*p* = 0.003−0.004), whereas the rotational accelerations of the dummy’s head was high during both impact events. When the results were compared to concussion injury risk thresholds (McIntosh et al., 2014) it demonstrated a high likelihood of concussion (LOC) injury to the dummy’s head in both impacts due to the large peak rotational accelerations observed (50–74% LOC > 1,747 rad/s^2^; 75% LOC > 2,296 rad/s^2^), and thus why both the initial impact and the subsequent ground impact must be considered in tackle research.

The linear accelerations from the player’s (tacklee’s) impact on the dummy’s head were not significantly different for any condition, which demonstrated the low protective value of the headgear worn against high impact linear forces. On the other hand, the rotational accelerations increased when headgear is worn when the dummy’s head struck the ground, compared with no headgear. This demonstrated that when a player’s head hits the ground following a tackle, rotational forces (torques) on the head may potentially be greater and therefore increase the risk of head injury when wearing headgear. It is possible that this increase in rotational forces was due to a greater moment arm about the axis of rotation. When the dummy strikes the ground headfirst, with the centre of mass being in the centre of the body, the axis of rotation then occurs at the less-rigid neck. If headgear is worn, the moment arm is increased by the thickness of the headgear, thus increasing the perpendicular distance from the axis of rotation, therefore increasing the torque of the impact. While this is a seemingly small alteration to the nature of the forces acting on the head, it was found to be consistently higher across the trials (*p* < 0.05). Given the susceptibility and vulnerability of the brain to injuries caused by movement, however small, the reason for this increase in rotational forces should be considered in further research and in the design of more effective headgear for the wearer.

Unexpectedly there was a significant reduction of linear acceleration values at the tackler’s shoulder when running at the very-fast (21 km/hr) speed for the headgear trials. The peak linear acceleration measured during the shoulder-head (of the tackle dummy) was lowest for the professional-level headgear (11.44 g), representing a reduction of −13% (−1.75 g) when compared to no headgear. For the club-level headgear the peak linear acceleration was reduced by –7% (−0.96 g) when compared to no headgear. This may indicate that at higher speeds, some cushioning is provided to the shoulder of the tackler by the opponent’s headgear, and that the level of cushioning is specific to the material properties of the worn headgear. Offensive players may therefore feel less of an impact on their own shoulder when tackling at the higher speed.

The findings of this study provide further evidence on the lack of effectiveness of headgear in rugby play. Further this study also revealed that in on-field conditions such as when a player is knocked over and impacts the ground with their head, wearing headgear can increase the rotational acceleration of the head and may therefore consequently increase the rotational forces experienced by the brain. The intended purpose of headgear is to protect the head by reducing the likelihood and magnitude of injury (McIntosh et al., 2003). Headgear has been reported to protect against superficial injuries on the outside of the head, with players wearing headgear to avoid perichondrial hematoma (“cauliflower ear”) (Marshall et al., 2005; Schneider et al., 2017). However, to have headgear not significantly reduce impact to the head and potentially increase rotational forces, is likely to have large ramifications on the head injury incidence in the sport. If headgear does lead to an athlete experiencing higher rotational forces in a game setting this could lead to a problematic scenario for those with a history of concussion. Research has shown that previously concussed (within the previous 12-month period) rugby athletes had higher head acceleration during tackles (Bussey et al., 2019). Hence, if an athlete suffers a concussion, and upon their return to training or play is wearing headgear to help prevent or reduce the severity of future concussions, the headgear may be having the opposite effect and could be harmful to their health.

Based on the findings, this new protocol can be used to investigate alternative headgear materials and designs to better attenuate impact forces to the head. The design of rugby headgear is restricted as the thickness is regulated and controlled by the rules of the game (World Rugby). However, different materials and designs should be investigated for their efficacy of protection. Future research investigating headgear materials could investigate Elastometric Microlattice Impact Attenuators that are classed as “intelligent materials”, that combine controlled buckling, viscous dissipation of force, and efficient adaptability of density (Clough et al., 2019). An example of this is the patented D3Ò material, that is being used most in body armour (Sun et al., 2012).

### Strengths and limitations of this study

This study has pioneered the integration of human tackling into the evaluation of rugby headgear. This provides an increase in the context validity of the results obtained, as opposed to testing with a single linear force applied by a machine in a laboratory. While this research was done in a laboratory, this method maintains ecological validity by ensuring as realistic a gameplay-like tackling as possible. A unique interplay of linear and rotational force from the running shoulder was applied to the dummy’s head that has not been quantified before. This was a more accurate test of the headgear as it was more representative of what the headgear would be subjected to in a rugby game. This study was the first to gauge the impact accelerations (linear, rotational) when someone’s head impacts the ground after being tackled. However, some of the results for the *very-fast-speed* tests should be treated with caution due to the poor inter-trial reliability results shown for the peak linear acceleration measure of the player’s shoulder during the tackle impact. This shows that more trials may need to be measured for tackles performed from running approaches at *very-high-speeds*.

Only one tackle scenario, including the head impact location, was tested in this study. The dummy was a free-standing model that represented a stationary ball carrier (opponent) with the front-on tackle occurring from the side. The tackler would be in the behind (blind spot)-to-peripheral visual field of the ball carrier (Garraway et al., 1999). This tackle collision scenario where the shoulder high tackle is executed, represents an arguably worst-case type of scenario for the ball carrier. That is also because there is a large differential between the speed of the tackling player and their ball carrying opponent, since the player with the lowest momentum has a higher risk of injury (Garraway et al., 1999). According to Walsh et al. (2018) the neck stiffness of the model may present a biased response for the acceleration components, but not for the resultant acceleration magnitudes (linear, angular) for shorter duration impacts. That bias due to the neck stiffness of the tackle dummy is consistent throughout the experimental tests. This methodological consistency ensures that relative differences between equipment (*e.g.*, headgear) can be accurately assessed, even if absolute values may differ from those experienced by human players. The bias of the neck stiffness of the tackle dummy likely has a greater impact when the findings of this study were compared to match-play classification systems for LOC. Therefore, a cautious approach to those contextual comparisons is needed.

The controlled nature of the study to minimize injury risk to tackling players, required players to perform the tackles in a laboratory environment. Some changes in behaviour and technique could be expected that would be different to the tackle techniques in a game on a grass field with a real opposing player rather than a dummy. Players could have tackled harder or softer in a “real” game, compared to tackling the dummy. This is also because the tackle dummy was a stationary and inert ball-carrying opponent with a lower mass (16.60 kg) and base of support. It should also be noted that participants of the study were male only, and we recognise that results may differ for female players.

Whilst studies such as ours contribute to increased understanding of tackle biomechanics, a conclusive threshold for concussion injury (Romeu-Mejia, Giza & Goldman, 2019) remains undetermined due to the multitude of intrinsic and extrinsic factors that contribute to whether an inciting event leads to injury (Meeuwisse et al., 2007). Sensors used to identify inertial forces present in high-speed tackles can only measure the more global parts of the mechanical-load response pathway model that is applicable in injury research, as it is unable to assess the physiological responses occurring within the brain tissue (Kalkhoven et al., 2021). Inputting the data from testing with this new method into strain-based brain models (Ji et al., 2015; Fortington et al., 2024; Stitt et al., 2024) may provide more holistic evaluations of impact conditions and brain strain related to injury.

## Conclusions

This study demonstrated, for the first time, a protocol of a player tackling a test dummy at gameplay speeds to enable measurements of player and dummy head linear and rotational accelerations at initial to ground impact during dangerous rugby high tackle technique of the players shoulder hitting the dummy head. The protocol was trialled using one impact location (left side of the head), two approach velocities and three headgear conditions (control, two headgear models). The headgear was not effective at reducing linear or rotational (angular) accelerations. In contrast the headgear increased impact head accelerations when contacting the ground. That the method demonstrated here can detect valuable information in predictable ways demonstrates its use in sports concussion research.

##  Supplemental Information

10.7717/peerj.20953/supp-1 Supplemental Information 1 Participant results for the tackle impacts on the instrumented dummy Headgear conditions; NH, no headgear; H1, club-level headgear; H2, professional-level headgear.
Approach (closing) velocity; 15, 15 kph; 21, 21 kph.
IMU location; P, participant’s tackling side; B, BOB instrumented dummy head.
Measurement; Accel, linear acceleration in g; RotA, rotational acceleration in rad/s2.
Impact event; 1, shoulder-head collision; 2, head-ground collision.

## Additional Information and Declarations

### Data Availability

The following information was supplied regarding data availability: The raw data is available in the Supplemental File.
